# The influence of male dominance in female *Anastrepha curvicauda* mate selection

**DOI:** 10.1038/s41598-021-85823-0

**Published:** 2021-03-18

**Authors:** Nancy Natividad Salmerón-Muñiz, René Arzuffi, Norma Robledo-Quintos, Alfredo Jiménez-Pérez

**Affiliations:** 1 Universidad Autónoma de Guerrero, Escuela Superior de Ciencias Naturales, Chilpancingo de los Bravo, 39105 Guerrero, Mexico; 2 Centro de Desarrollo de Productos Bióticos (CEPROBI) del Instituto Politécnico Nacional, Calle Ceprobi No. 8, San Isidro, 62731 Yautepec, Morelos Mexico

**Keywords:** Agroecology, Behavioural ecology

## Abstract

Males of the papaya fruit fly, *Anastrepha curvicauda* Gerstaecker (former *Toxotrypana curvicauda*), defend a papaya fruit from rivals and males release their sex pheromone to attract and mate with females and offer them an oviposition site. While some aspects of the biology of *A. curvicauda* are known, such as its reproductive biology, its sex pheromone, and host selection, there is currently no information on the species mate selection process. This paper describes the precopulatory mating behavior of *A. curvicauda* and elucidates how intrasexual selection affects the mate selection process. We studied the precopulatory mating behavior of dominant and subordinate males and ethograms were devised. The effect of hierarchy was studied in non-choice and choice experiments. Male’s repertoire includes 15 behavioral elements, 12 precopulatory, one mating, and two postcopulatory (tandem and encounter). In non-choice experiments, dominant and subordinate males were accepted by females, but when females had the opportunity to choose among males, dominant males were significantly preferred over subordinate ones. The presence of a rival male modified the courting behavior of males and agonistic behavior among males was observed before and during mating.

## Introduction

Dominance is a social attribute among two individuals, and it relates to the place of an individual among its conspecifics. Dominant individuals are recognized by their agonistic behavior and level of aggressivity, and the losing rivals are known as subordinates. Dominance could be high or low creating a hierarchy^1^. Hierarchy is a temporal attribute between two individuals in a social context. Dominant individuals tend to be stronger, healthier and have an easier path to food, shelter, or reproduction than subordinated ones^1,2^. Dominance behavior is widespread among animals and provokes variation in the ability of an individual to access food, sexually mature mates, and other limiting resources^1,3^.

Courtship is a two-way interaction process where the males perform different behaviors to be accepted by the females. Females use courtship behaviors to evaluate potential males as they may be indicative of male quality^4^ and this courtship may end at any point as one individual may withdraw or fly away at any moment and courting males also may deal with potential attacks by rival^5^.

Females may benefit from selecting the best male either by obtaining indirect (genetics) benefits (an increase in fitness like those suggested by the sexy son and good genes hypothesis)^6,7^ or direct benefits like an increase in fecundity and fertility^7,8^. For example, choosy females of *Anastrepha fraterculus* (Wiedemann) increase their fecundity and fertility by mating males exposed to guava volatiles^9^. Female choosiness may vary from mate to mate, during a mating season^10–12^, and in time and space^5^. This change in female choosiness may increase the cost of searching for mates and the risk of failure to mate but may gain direct and indirect genetic benefits from mating with high-quality males^13^ if these genetic benefits correlate with males’ competitive ability^5^, yet individual female preference may diverge from these general statements^4,13^.

Tephritidae courtship behavior can be divided into three stages: (a) males defend a territory from other males (agonistic behavior) and release a pheromone, acoustic cues (e.g. wing vibration) and visual signs to lure females in, (b) nearby females land in the males’ territory and (c) males detect females and intent mating^14^. A female’s decision to mate with a particular male is influenced by the male’s attributes (physical or behavioral) which may indicate a better genetic quality or reproductive potential^15,16^ and the status (dominant or subordinate) a male has in the hierarchy among the males^1^.

Many Tephritidae, like *Ceratitis capitata* Loew*, A. fraterculus*, *Bactrocera cucurbitae* Coquillett, and *B. tryoni* (Froggatt) form leks as part of their mating systems^17–19^. These leks congregate many males in a specific area where most copulations occur. Intrasexual competition is intense and reproductive success varies as many males fail to mate and a few males mate several times^20,21^.

Some Tephritidae like *Rhagoletis pomonella (Walsh), R. indifferens Curran, R. suavis (Loew),* and *Anastrepha curvicauda* Gerstaecker (former *Toxotrypana curvicauda*, the papaya fruit fly), use host plants as mating grounds and do not form leks^17,22^. *A. curvicauda* males are territorial and defend a papaya fruit (oviposition site) from rival males, where they emit sex pheromone to lure females in^22,23^ and mate^24,25^. Intrasexual competition in non-forming leks species is less severe than in forming leks species and many males achieve mating^25^.

*Anastrepha curvicauda* individuals are relatively large (2–2.5 cm long) and easy to collect from infested papaya fruit. Females are sexually mature at 4 days old^26^, while newly emerged males have been observed courting females and may achieve mating. Both genders may mate more than once under lab conditions. Its mating system is resource-based^27^ and includes visual cues, sound, sex pheromone, courtship^28–30^, and agonistic behavior that influences the capability of males to perform a courtship, and the choosiness of females. This agonistic behavior among males in the field has been described previously^22^. In brief, males face each other, raise the thorax and abdomen with lateral movements, and touch each other’s second legs, wing fan and touch their opponent with the forelegs. Sexual selection theory^2^ suggests that males successfully defending a territory have more chances to achieve mating. In other words, males able to dominate rival males would have a reproductive advantage.

Based on field reports^22,24^ and our empirical observations at our experimental papaya grove, we hypothesized: (1) agonistic behavior among males establishes a hierarchy where the dominant male gains access to females more often than subordinate males, (2) males may modify their courtship repertoire and effort in the presence of a rival male, (3) females mate preferentially with dominant males over subordinate ones, and (4) males not selected by females for mating would try to interrupt mating pairs. This paper reports qualitative and quantitative findings on how male courtship and successful mating are affected by the presence of a rival male. Understanding intrasexual selection and the mating sequences could help to understand mate choice, sexual selection and provide information for the development or improvement of behavioral based control strategies.

## Results

### Courtship repertoire

**Figure 1:**
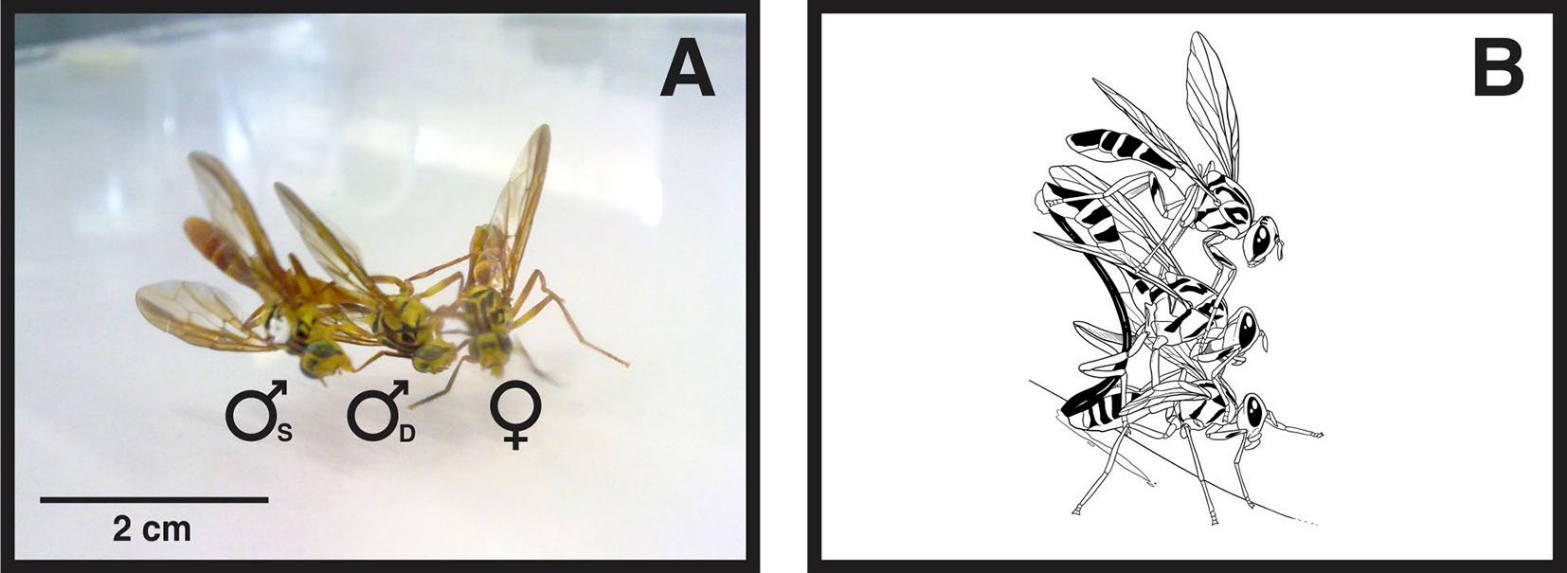
Tandem behavior of *Anastrepha curvicauda* (**A**) a female (♀) is mounted by a dominant male (♂_D_) and this male is mounted by a subordinated male (♂_S_) and (**B**) graphic representation of the tandem behavior.

A total of 12 different courtship behavioral elements (premating) were recorded and in all cases where mating was achieved (Table 1). Further behaviors were identified in the choice experiments; the mating pair were mounted by the rival male (a behavior called tandem or TA, Fig. 1A,B), the fight between males occurred frequently (agonistic behavior mentioned hereafter as Encounter or E) and withdraw from the encounter (W).

**Table 1:** Courtship behavioral elements of *Anastrepha curvicauda* (7–8 days old) males in choice and non-choice experiments.

	Behavioral elements	Description
Behaviors displayed in choice and non-choice experiments	OMF	Orientation and movement towards the female
	S	Swing of the body laterally
	SWF	Swing of the body and wing fanning
	WAF	Walks around the female
	MCA	Moves in a circle on its axis
	TFF	Touches female with forelegs
	RA	Raise the abdomen
	WF	Wing fanning (with or without abdomen movement)
	SOTF	Still oriented towards female
	CF	Chases the female
	LDCF	Lose direct contact with female
	MF	Mounts the female
	MA	Mating
Behaviors displayed only in the choice experiments	TA	Tandem
	E	Encounter
	W	Withdraw from the encounter

**Figure 2:**
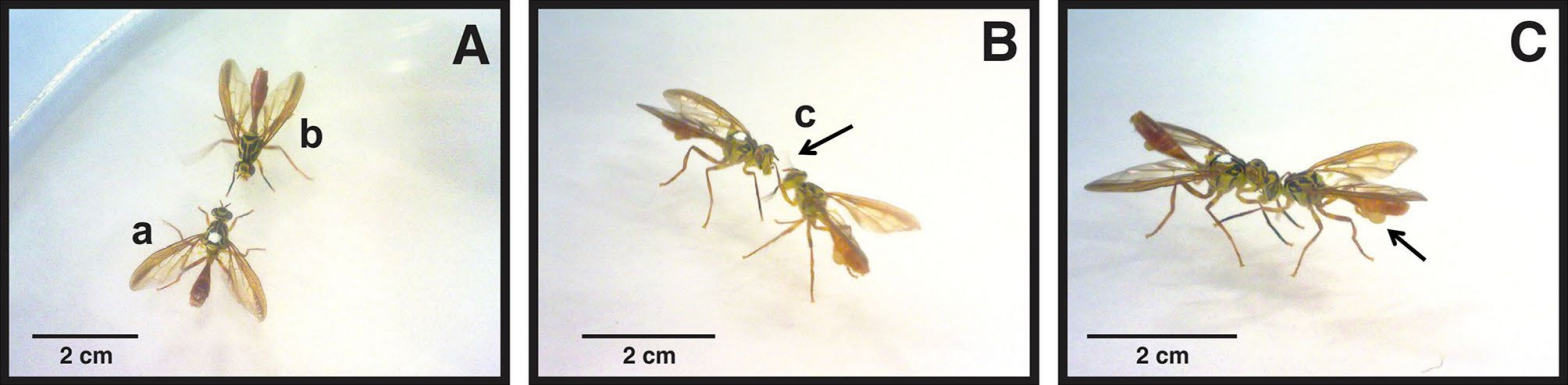
Agonistic behaviors displayed by *Anastrepha curvicauda*. (**A**) Threat: male (b) is threatening male (a). Male (b) raises its abdomen at a sharp angle, folds back its wings, and waves its middle legs up and down making a characteristic sound, (**B**) Attack: the male on the right is striking the other male in the head with his forelegs. The arrow indicates the forelegs hitting the head of the rival, (**C**) Fight between males. Arrow indicates expanded pleural pouches.

In choice experiments, on few occasions, we observed that shortly after the dominant male dismounted the female, the subordinate male would court the female and attempt mating without success. Even more, the dominant male would threaten and chase away the other male. This may include a direct threat, attack, or fight between males (Fig. 2A–C, respectively).

### Effect of hierarchy on courtship behavioral elements

#### Non-choice experiments

**Figure 3:**
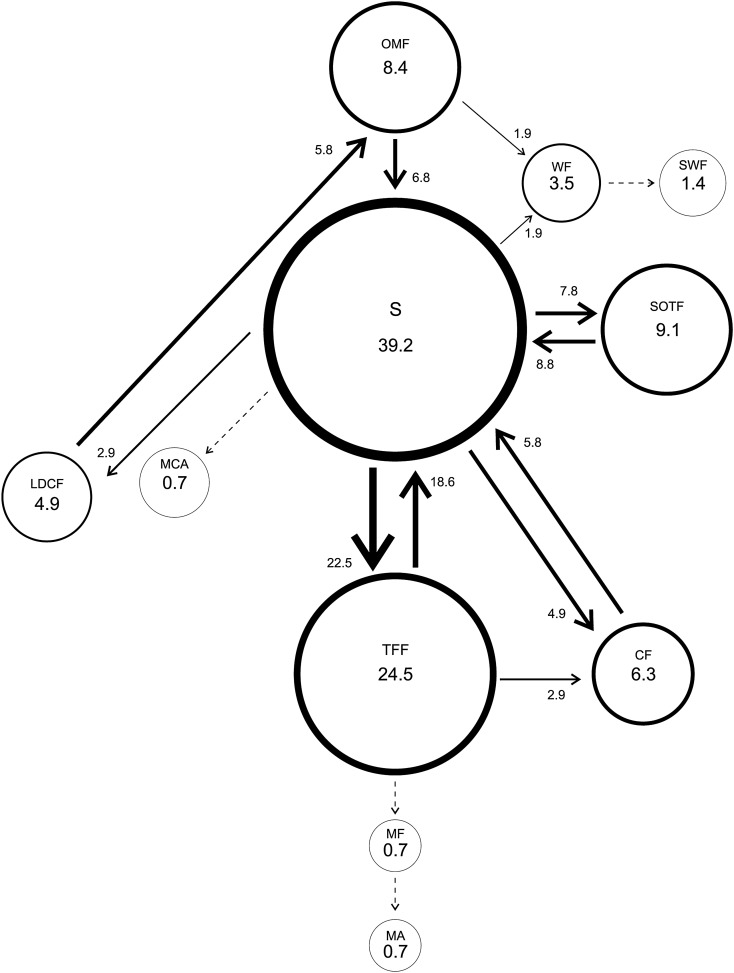
Courtship behavior of *Anastrepha curvicauda* dominant 7–8 days old males caged with a 5–7 days old virgin female. Circles represent behavioral elements and arrows transitions. Circle size and line thickness are proportional to their percentage values. Dotted lines represent transitions with a median < 1. See Table 1 for abbreviations.

Dominant males performed 11 different courtship behavioral elements with “Swing” the most frequent (39.2%) and “Moves in a circle on its axis”, “Mount the female” and “Mating” the less frequent (0.7%) ones. The “Swing-Touch the Female with the Forelegs” transitions were the most frequent with 22.5%, while “Swing-Moves in a circle on its axis”, “Wing Fanning-Swing of the body and Wing fanning”, “Touch the Female with the Forelegs-Mount the Female” and “Mount the Female-Mating” with values below 1% (Fig. 3).

**Figure 4:**
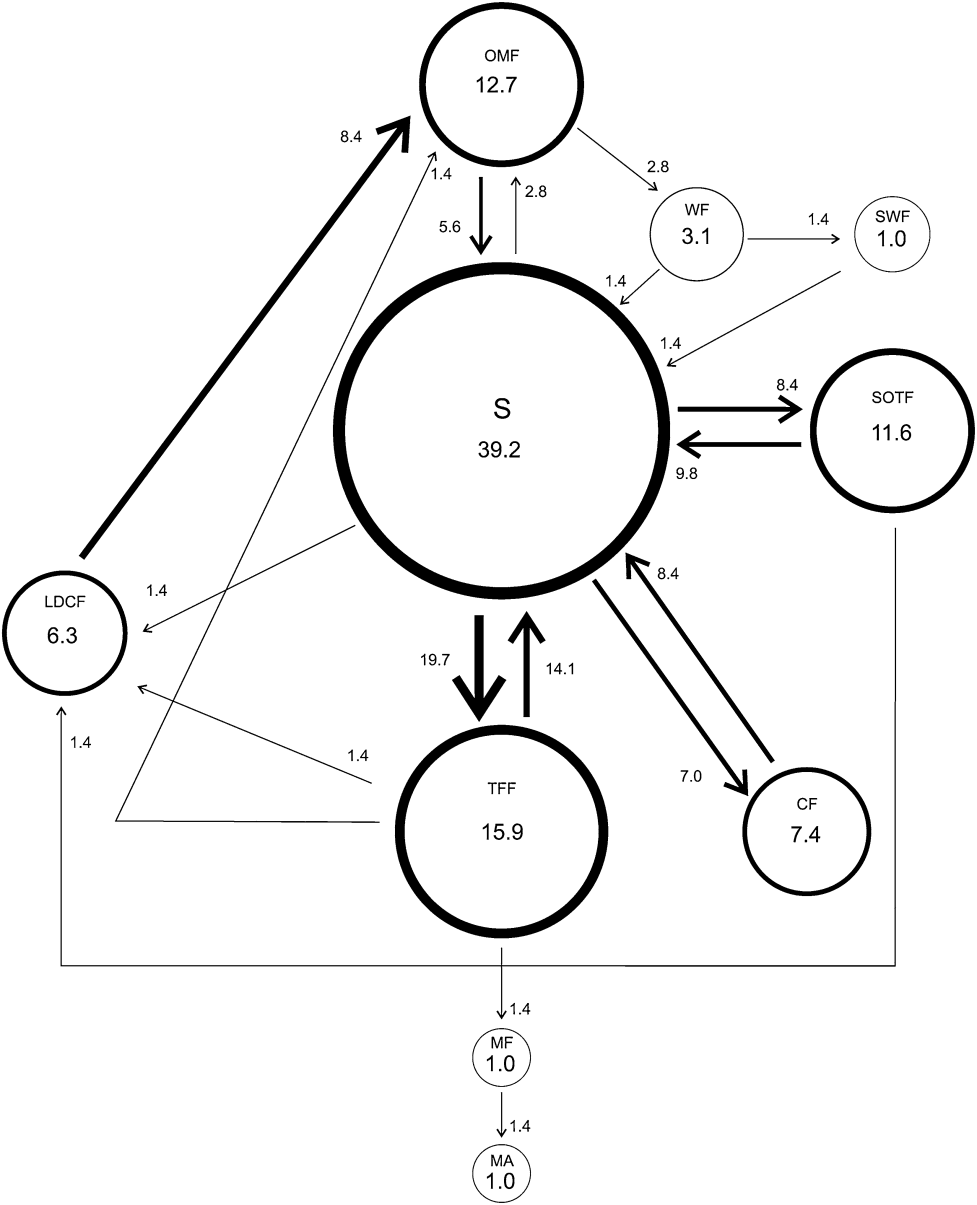
Courtship behavior of *Anastrepha curvicauda* subordinate 7–8 days old males caged with a 5–7 days old virgin female. Circles represent behavioral elements and arrows transitions. Circle size and line thickness are proportional to their percentage values. Dotted lines represent transitions with a median < 1. See Table 1 for abbreviations.

Subordinate males showed a total of 10 different courtship behavioral elements been “Swing” the most frequent (39.2%) and “Swing of the body with Wing Fanning” and “Mount the Female” and “Mating” the less frequent with 1.0%. The most frequent transition was “Swing-Touch Females with Forelegs” with 19.7% while “Wing Fanning-Swing of the body laterally”, “Wing Fanning-Swing of the body and Wing Fanning”, “Swing of the body and Wing Fanning-Swing of the body laterally”, “Touches Female with Forelegs-Mount the Female”, “Swing of the body laterally-Withdraw”, “Touch Female with Forelegs-Orientation and Movement towards the Female”, “Touch the Female with Forelegs-Withdraw”, “Still Oriented Towards Female-Withdraw” and “Mount the female-Mating” with 1.4% (Fig. 4).

Dominant males performed “Touches Female with Forelegs” (Table 2) and the transition “Touches Female with Forelegs-Chases the Female” significantly more times than subordinates (Table 3).

**Table 2:** Frequencies of courtship behavioral elements of *Anastrepha curvicauda* dominant and subordinate males (7–8 days old) caged with a virgin 5–7 days old female.

Behavioral elements	Male status		P
	Dominant	Subordinate	
OMF	6.00 < 12.00 < 29.00	5.00 < 12.00 < 32.00	0.983
S	29.00 < 56.00 < 82.75	9.75 < 37.00 < 82.00	0.520
SWF	1.00 < 2.00 < 3.00	0.25 < 1.00 < 4.25	0.656
MCA	0.00 < 1.00 < 3.00	0.00 < 0.00 < 3.50	0.752
TFF	17.50 < **35.00** < 71.50	5.00 < **15.00** < 33.50	0.042
WF	1.25 < 5.00 < 11.75	2.00 < 3.00 < 4.00	0.738
SOTF	5.00 < 13.00 < 20.50	1.00 < 11.00 < 24.00	0.693
CF	5.25 < 9.00 < 31.25	1.00 < 7.00 < 13.50	0.307
LDCF	2.00 < 7.00 < 16.50	1.25 < 6.00 < 12.00	0.739
MF	0.00 < 1.00 < 1.00	0.00 < 1.00 < 1.00	0.833
MA	0.00 < 1.00 < 1.00	0.00 < 1.00 < 1.00	0.728

Non-choice experiment.

Reported values are Q1 < Median < Q3 (n = 15). Medians in bold in the same row are significantly different (Mann–Whitney test, p > 0.05). See Table 1 for abbreviations.

**Table 3:** Frequencies of transitions between the courtship behavioral elements of *Anastrepha curvicauda* dominant and subordinate (7–8 days old) males caged with a virgin 5–7 days old female.

Transition	Male status		p
	Dominant	Subordinate	
OMF-S	4.00 < 7.00 < 16.00	2.00 < 4.00 < 22.75	0.851
OMF-WF	1.00 < 2.00 < 4.00	1.00 < 2.00 < 3.75	0.784
S-OMF	0.00 < 1.00 < 2.75	1.00 < 2.00 < 9.00	0.089
S-TFF	12.25 < 23.00 < 36.75	4.25 < 14.00 < 23.00	0.078
S-WF	0.00 < 2.00 < 2.00	0.00 < 0.00 < 1.00	0.142
S-SOTF	4.00 < 8.00 < 16.00	0.25 < 7.00 < 17.75	0.708
S-CF	1.50 < 5.00 < 16.50	0.25 < 5.00 < 10.75	0.602
S-LDCF	0.00 < 3.00 < 5.25	0.00 < 1.00 < 4.75	0.865
SWF-B	0.00 < 1.00 < 2.00	0.00 < 1.00 < 1.75	0.948
TFF-OMF	0.00 < 1.00 < 3.75	0.00 < 1.00 < 2.00	0.776
TFF-S	8.00 < 19.00 < 29.25	2.00 < 10.00 < 15.75	0.081
TFF-CF	1.00 < **3.00** < 5.75	0.00 < **0.00** < 1.75	0.024
TFF-LDCF	0.25 < 1.00 < 3.50	0.25 < 1.00 < 2.00	0.698
TFF-MF	0.00 < 1.00 < 1.00	0.00 < 1.00 < 1.00	0.981
WF-S	0.25 < 1.00 < 2.75	1.00 < 1.00 < 2.00	0.948
WF-SWF	0.00 < 1.00 < 1.00	0.00 < 1.00 < 2.00	0.406
WF-CF	0.00 < 1.00 < 1.00	0.00 < 0.00 < 0.75	0.155
SOTF-LDCF	0.00 < 1.00 < 1.00	0.00 < 1.00 < 1.75	0.982
CF-S	1.50 < 6.00 < 21.00	1.00 < 6.00 < 11.50	0.560
LDCF-OMF	2.00 < 6.00 < 14.50	1.00 < 6.00 < 13.00	0.787
MF-MA	0.00 < 1.00 < 1.00	0.00 < 1.00 < 1.00	0.728

Non-choice experiment.

Reported values are Q1 < Median < Q3 (n = 15). Medians in bold in the same row are significant different (Mann–Whitney test, p > 0.05). See Table 1 for abbreviations.

#### Choice experiments

**Figure 5:**
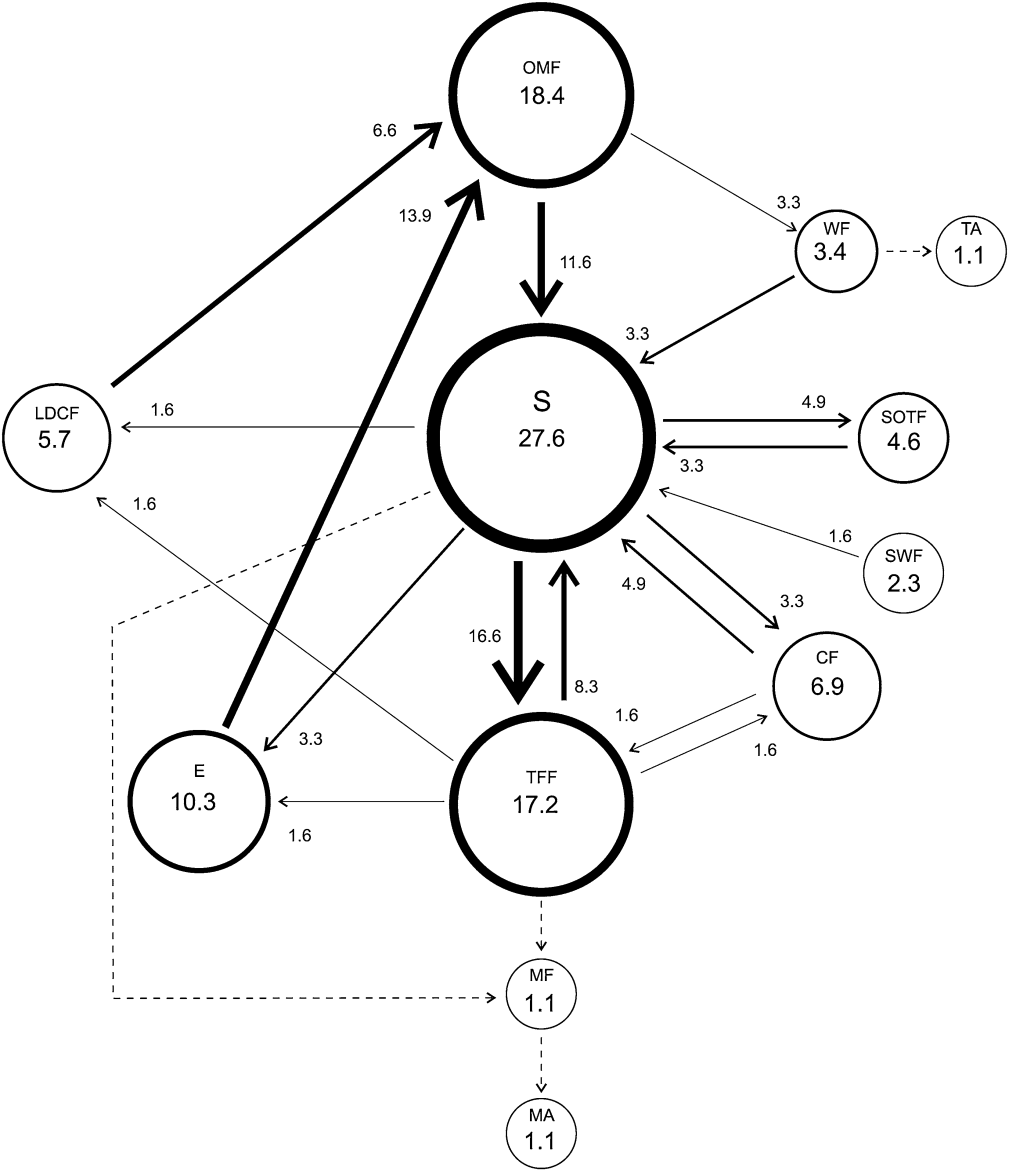
Courtship behavior of *Anastrepha curvicauda* dominant 7–8 days old males caged with a 5–7 d old virgin female and a subordinated male. Choice experiment. Circle size and line thickness are proportional to their percentage values. Dotted lines represent transitions with a median < 1. See Table 1 for abbreviations.

**Figure 6:**
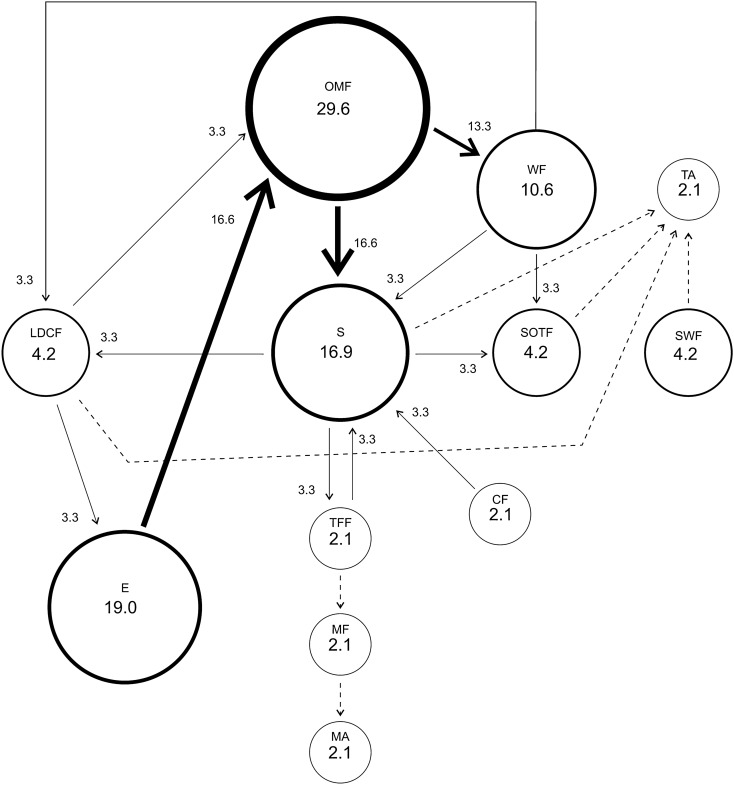
Courtship behavior of *Anastrepha curvicauda* subordinate 7–8 days old males caged with a 5–7 days old virgin female and a dominant male. Choice experiment. Circle size and line thickness are proportional to their percentage values. Dotted lines represent transitions with a median < 1. See Table 1 for abbreviations.

Twelve behavioral elements were observed in their courtship when a dominant and a subordinate male were caged simultaneously with a 5–7 days old virgin female (Figs. 5 and 6). The most frequent courtship behavioral element for the dominant males was “Swing of the body laterally” (27.6%) and “Orientation and Movement towards the Female” (29.6%) for the subordinate males. The most frequent transition of the dominant males was “Swing of the body laterally-Touches Female with Forelegs” (16.6%) and for subordinate males were “Orientation and Movement towards the Female-Swing of the body laterally” and “Encounter- Orientation and Movement towards the Female” both with 16.6%.

The least frequent transitions behavioral elements for the dominant males were “Wing Fanning-Tandem”, “Swing of the body laterally-Mount the Female”, “Touches Female with the Forelegs-Mount the Female” and “Mount the Female-Mating” (Fig. 5). For subordinate males, the least frequent transitions were “Swing of the body laterally-Tandem”, Still Oriented to the Female-Tandem”, Swing of the body with Wing Fanning-Tandem”, “Lose Direct Contact with Female-Tandem”, “Touches the Female with the Forelegs-Mount the female” and “Mount the Female-Mating” with values below 1% (Fig. 6).

“Orientation and Movement towards female” and “Orientation and Movement towards the Female-Swing of the body” were the most frequent behavioral element transition for subordinate males because the dominant male would court the female most of the time and the subordinate males remained away from the dominant male and the female. When the subordinate male had the opportunity to get close to the female, he performed “Orientation and Movement to the female” and swing his body, but the dominant male would chase him away either by threat, punching or pushing or by courting the female and moving her away from the subordinate male.

When comparing the courtship behavioral elements frequency, dominant males performed “Touches Female with Forelegs” significantly more times than subordinate males (Table 4). Similarly, dominant males transited “Touches Female with Forelegs-Swing of the body laterally” significantly more times than subordinate males (Table 5).

**Table 4:** Frequencies of courtship behavioral elements of *Anastrepha curvicauda* dominant and subordinate (7–8 days old) males caged with a virgin 5–7 days old female.

Behavioral elements	Male status		P
	Dominant	Subordinate	
OMF	2.75 < 16.00 < 40.50	2.00 < 14.00 < 26.00	0.479
S	7.50 < 24.00 < 33.25	1.00 < 8.00 < 22.50	0.130
SWF	0.00 < 2.00 < 4.75	0.00 < 2.00 < 2.75	0.477
TFF	5.50 < **15.00** < 22.00	0.00 < **1.00** < 10.25	0.013
WF	2.00 < 3.00 < 16.00	2.00 < 5.00 < 9.50	0.787
SOTF	1.25 < 4.00 < 7.75	1.25 < 2.00 < 5.00	0.326
CF	0.25 < 6.00 < 16.75	0.00 < 1.00 < 6.50	0.103
LDCF	0.25 < 5.00 < 10.75	1.00 < 2.00 < 8.75	0.439
E	1.50 < 9.00 < 21.50	1.50 < 9.00 < 21.50	0.983

Choice experiment.

Reported values are Q1 < Median < Q3 (n = 15). Medians in bold in the same row are significantly different (Mann–Whitney test, p > 0.05). See Table 1 for abbreviations.

**Table 5:** Frequencies of transitions between the courtship behavioral elements of *Anastrepha curvicauda* dominant and subordinate (7–8 days old) males caged with a virgin 5–7 days old female.

Transitions	Male status		P
	Dominant	Subordinate	
OMF-S	2.00 < 7.00 < 17.75	0.00 < 5.00 < 13.50	0.514
OMF-WF	1.00 < 2.00 < 7.75	1.00 < 4.00 < 7.75	0.676
S-TFF	1.25 < 10.00 < 15.25	0.00 < 1.00 < 5.00	0.098
S-SOTF	0.25 < 3.00 < 5.00	0.00 < 1.00 < 1.75	0.114
S-CF	0.00 < 2.00 < 6.50	0.00 < 0.00 < 2.75	0.140
S-LDCF	0.00 < 1.00 < 4.50	0.00 < 1.00 < 4.25	0.728
S-E	0.00 < 2.00 < 5.25	0.00 < 0.00 < 5.50	0.743
SWF-S	0.00 < 1.00 < 1.00	0.00 < 0.00 < 0.75	0.108
TFF-S	1.50 < **5.00** < 9.00	0.00 < **1.00** < 3.50	0.016
TFF-CF	0.00 < 1.00 < 2.00	0.00 < 0.00 < 1.00	0.267
TFF-LDCF	0.00 < 1.00 < 2.75	0.00 < 0.00 < 0.75	0.084
TFF-E	0.00 < 1.00 < 3.75	0.00 < 0.00 < 2.50	0.351
WF-S	0.25 < 2.00 < 2.75	0.00 < 1.00 < 1.00	0.077
WF-SOTF	0.00 < 0.00 < 0.00	0.00 < 1.00 < 1.00	0.062
WF-LDCF	0.00 < 0.00 < 1.00	0.00 < 1.00 < 1.00	0.517
CF-S	0.25 < 3.00 < 6.50	0.00 < 1.00 < 3.00	0.140
CF-TFF	0.00 < 1.00 < 1.00	0.00 < 0.00 < 0.00	0.066
LDCF-OMF	0.00 < 4.00 < 7.50	0.25 < 1.00 < 4.50	0.423
LDCF-E	0.00 < 0.00 < 2.75	0.00 < 1.00 < 2.00	1.000
E-OMF	1.25 < 8.00 < 17.00	1.50 < 5.00 < 15.00	0.662

Choice experiment.

Reported values are Q1 < Median < Q3 (n = 15). Medians in bold in the same row are significantly different (Mann–Whitney test, p > 0.05). See Table 1 for abbreviations.

### Effect of hierarchy on courtship variables and mating

No differences were observed when comparing courtship variables among the dominant and subordinate male groups in the non-choice experiments (Table 6). In the same way, mating percentages achieved by the dominant (66.6%) and the subordinate (60%) males were not different (χ^2^ = 0.536, p = 0.464).

**Table 6:** Duration of courtship variables of *Anastrepha curvicauda* dominant and subordinate (7–8 days old) males.

Variables	Male status		P
	Dominant	Subordinate	
Courtship vigor index	0.31 < 0.59 < 0.69	0.28 < 0.48 < 0.65	0.37
Latency to courtship (s)	21.50 < 157.00 < 312.75	87.75 < 267.00 < 654.25	0.21
Courtship length (s)	841.2 < 1197.00 < 2033.7	120.7 < 962.00 < 1647.7	0.34
Latency to mating (s)	1347.0 < 3037.0 < 3600.0	502.2 < 2910.0 < 3600.0	0.94

Non-choice experiments.

Reported values are Q1 < Median < Q3 (n = 15). Mann–Whitney test, p > 0.05.

However, when a female was caged with both males, dominant males registered a significantly lower courtship vigor index and achieved mating earlier than subordinate males (Table 7). Females mated more with dominant (85.71%) than with subordinate (14.28%) males (χ^2^ = 100.82, P < 0.001).

**Table 7:** Courtship variables of *Anastrepha curvicauda* dominant and subordinate (7–8 days old) males.

Variables	Male status		P
	Dominant	Subordinate	
Courtship vigor index	0.18 < **0.27** < 0.39	0.012 < **0.060** < 0.16	0.001
Latency to courtship (s)	23.25 < 134.00 < 225.00	61.75 < 223.00 < 430.75	0.178
Courtship length (s)	174.00 < 762.00 < 977.25	60.00 < 238.00 < 590.75	0.074
Latency to mating (s)	1144.7 < **3600.0** < 3600	3600 < **3600.0** < 3600	0.029

Choice experiments.

Reported values are Q1 < Median < Q3 (n = 15). Medians in bold in the same row are significantly different (Mann–Whitney test, p > 0.05).

Regardless that hierarchy was well established among males before the experiment, males engaged in a continuous and prolonged fight for gaining access to the female, for this reason, some dominant males failed to achieve mating.

## Discussion

Males of *A. curvicauda* performed a total of 15 behavioral elements, 12 are related to courtship, one mating, and two post-copulatory. These behaviors had been reported previously^28,31^ in papaya groves. They described precopulatory behavioral elements like “Swing of the body”, “Swing of the body and Wing Fanning”, “circle the female”, “touches Female with Forelegs”, “Raise the Abdomen” and “Wing fanning”. However, the following behavioral elements are reported for the first time: “Moves in circle on its axis”, “Orientation and Movement towards the Female” and “Chase the Female”. In comparison, the courtship behavior of *Anastrepha ludens* (Loew), a lekking fruitfly with a short sexual activity period, includes five behaviors: calling by males, females arrive at a leaf, the male orients toward the female and stops calling, one or both insects get closer in a “vis à vis” position and finally the male mounts the female^32^. This difference in the number of behaviors involved in the ethogram could be related to the mating system. Leks may congregate up to 5–7 males where competition for females is severe. An elaborated courtship requires time increasing the probability that a rival male challenge the courting male and disrupt the potential mating couple. Our observations in an experimental papaya groove indicate that is very common to find an *A. curvicauda* male per papaya fruit while having 2 or more males perching in a papaya fruit is not so common as a conflict between males arises. This observation is in line with that of Landolt and Hendrick^22^ were papaya trees with two or three males per tree, males were observed in separated fruits on 76 and 66% of occasions.

In most cases, when two males were caged with a female, mating was preceded by agonistic behavior among males and some subordinate males prevented the dominant male from mating. In the behavioral element named “tandem”, the dominant male mounts the female and introduces his aedeagus into the female while the other male mounts the first male and unsuccessfully tries to dislodge him or introduce his aedeagus into the female. A few seconds after the dominant male finished copulating with the female, the subordinated male tried to copulate with the female but failed to mate.

In the single case where the subordinate male achieved mating, males had had more than 10 previous “Encounter” and the subordinate male had courted the female 7 times before mating. When the female accepted mating, the subordinate male was closer to the female and gained access to her. The dominant male mounted the mating couple (tandem behavior) and failed to separate the mating pair. Immediately, after mating, the dominant male mounted and copulated with the female. We did not establish if both males transferred an ejaculate, if dominant males transferred a better quality one and if there was any type of postcopulatory selection like reported for *C. capitata*^33^, *A*. *fraterculus*^34^, and *A. ludens*^35^. Non-winning males attacking or disturbing pairs in copula has been also reported in *A. ludens*^32^. We had noticed the tandem behavior during our observations at our papaya groove. The tandem behavior could serve the “losing” males in two ways: (a) to be close to a receptive female and (b) to disrupt the mating couple.

In non-choice experiments, the most conspicuous behavioral element and transitions for dominant and subordinate males were “Swing of the body laterally” and “Swing of the body laterally-Touches Female with forelegs”, respectively. However, subordinate males’ courtship ethograms indicated more transitions and with frequencies > 1 than that of dominant males indicating that subordinate males perform a more elaborate courtship to achieve mating. In contrast, dominant males touched females more frequently to achieve mating suggesting that touching increases their chances of mating. In *Musca domestica*^36^, *D. elegans* and *D. gunungcola*^37^ and *Lucilia sericata* (Meigen)^38^ touching transfer chemical cues (like cuticular hydrocarbons) which may facilitate conspecific and sexual identification^39^.

In choice experiments, when dominant males achieved mating, subordinate males most frequently performed the tandem behavior. Subordinate males may withdraw and move away from the female and still have an encounter with the dominant male (Withdraw-Encounter transition). Dominant males may court the female and if necessary, chase away any rival male (Swing of the body laterally -Encounter and Touches Female with Forelegs-Encounter transitions). These behaviors could be related to the territorial behavior described in the field^22^, where a male chases away any rival male who dares to land on the papaya fruit defended by him. Males defending a resource (the papaya fruit as oviposition place) and chasing away any rival male increases its chances to achieve mating^40^. Similar results were reported for *A. suspensa* (Loew)^41^, *Bactrocera oleae* (Rossi)^16^, and *Ragoletis completa* (Cresson) males^40^.

In our experiments, wing fanning frequency was similar in all cases, which is not in line with that reported for other Tephritidae. Males of *C. capitata*^42^, *Bactrocera dorsalis* (Hendel)^43^, and *A*. *fraterculus*^9^ who invested more time in wing fanning were more likely to achieve mating. Females may use wing fanning as a correlate of male health and vigor^16^.

Dominant males performed “Touches Female with Forelegs” and the transition “Touches Female with Forelegs—Swing of the body laterally” more frequently, which could be explained because these males get closer to the female during courtship and chase away the subordinate males. We do not know if by getting closer and more frequently to the females, *A*. *curvicauda* males receive cues from the females of their likelihood of achieving mating as reported in *Drosophila melanogaster* Meigen and *D*. *simulans* Sturtevant^44^, but it is worth investigating.

According to our results from the non-choice experiment, courtship variables were not affected by previous male-male encounters when dominant or subordinate males were caged with a female indicating that males may follow the same courtship behavioral elements to achieve mating. Females would accept mating with dominant or subordinate males if that is the only male available^10^. If there is any variance in the quality of an ejaculate provided by a dominant or subordinate male, females may accept mating with other males to increase their fitness. A 1.9:1 male:female ratio was reported at a papaya groove^24^ and a 1:1.26 male:female ratio at lab conditions^26^, therefore, female remating is highly probable as there is plenty of *A. curvicauda* males available at the papaya groove. However, when a dominant and a subordinate male were caged with a female, the dominant male would head for the female, court her, and achieve mating. Also, dominant males interrupted subordinate male courtship and chased the subordinate away. *Bactrocera oleae* males that have lost their first encounter would fight even harder on their next opportunity, scaling its level of aggressivity, so fight skills and its intensity are the results of previous experiences^45^. Our experiment design established a hierarchy between males with no female in the vicinity and 90 min later, tested the hierarchy previously established among males and included a virgin female in the cage. The presence of the female would bust the level of aggressiveness as males are competing to gain access to the female while competing among them, so our design not only gave the subordinate male a second chance to win a fight but also the presence of a virgin female increased the desire to win the contest and achieve mating. These behaviors may explain the reduction in dominant male courtship vigor index, latency to mating, and the reduction of matings observed in the choice experiment.

It has been reported that *A. curvicauda* larger males (50% heavier than light ones) achieved mating more frequently than small ones because the former males produce a louder approach song^29^. In our research, male weight varied no more than 10%, reducing a possible sound effect related to male size affecting our trials.

Intrasexual selection in *A. curvicauda* males is the way to access females and, the hierarchy among males is specific for two particular males. Dominant males were always dominant, however, both dominant and subordinate males displayed agonistic behavior when competing for a female. Male courtship behavior is an established sequence. When more than one male court a female, dominant males reduced their courtship vigor index. Similarly, dominant males achieved mating earlier while most subordinate males failed to mate. When a male (dominant or subordinate) was caged with a female alone, females did not discriminate among dominant or subordinated males for mating as a similar number of matings were recorded for each male group. When males interacted between them and with a female, a significant number of females accepted the dominant males as partners. Fight among males for access to females may happen before or during mating, but no male guarded the female after mating.

We acknowledge that the behavior reported in this paper is a simplification of what may happen in the wild (intra and intersexual selection for both genders, a changing operational sex ratio to mention a few) where more than two males or females may fight for the opportunity to mate and both genders must deal with potential predators. However, we think this information is valuable as it is the first quantitative report on male courtship and the effect of agonistic behavior among males on *A. curvicauda* mate selection and expands our knowledge on dipteran courtship.

## Methods

### Insects

We obtained flies from infested papaya fruit according to the method previously described^26^. We used cylindrical plastic containers (500 mL capacity) with sterile soil as pupation substrate. Newly emerged flies (0 day) were weighed in an analytical Explorer Pro, Ohaus scale and contained individually in a 200 mL transparent cylindrical plastic to avoid any social interactions. Males and females were kept in separate rooms until needed to avoid odors (e.g. sex pheromones) between females and males. Flies were fed a 2% percent sugar solution as this fly does not require protein intake for egg production^46^. All insects were kept at 25 ± 2 °C and a 12:12 photoperiod. In all cases, male weight varied within 0.05 mg; male average weight was 42.7 mg and ESM = 0.70 mg^26^. Insects were used once and then discarded.

All experiments were performed at the time of day when most sexual activity occurs (11:30 to 16:00 h)^22,24^. Trials were filmed for 60 min with a Handycam 700× Sony video camera and were carried out under the aforementioned environmental conditions. All behaviors were recorded manually watching each video and writing down each behavior observed. A pilot experiment showed that 10 out of 12 females accepted mating within 60 min. Mating was defined as Landolt and Hendricks^17,25^ sensu lato and pairs remaining in copula for more than 10 min. When two males were involved in an experiment, one of them was randomly assigned to be marked on the dorsal part of the thorax with a tiny white dot. This white dot did not restrain its movements nor its probability of achieving mating (binomial test, p > 0.05).

**Figure 7:**
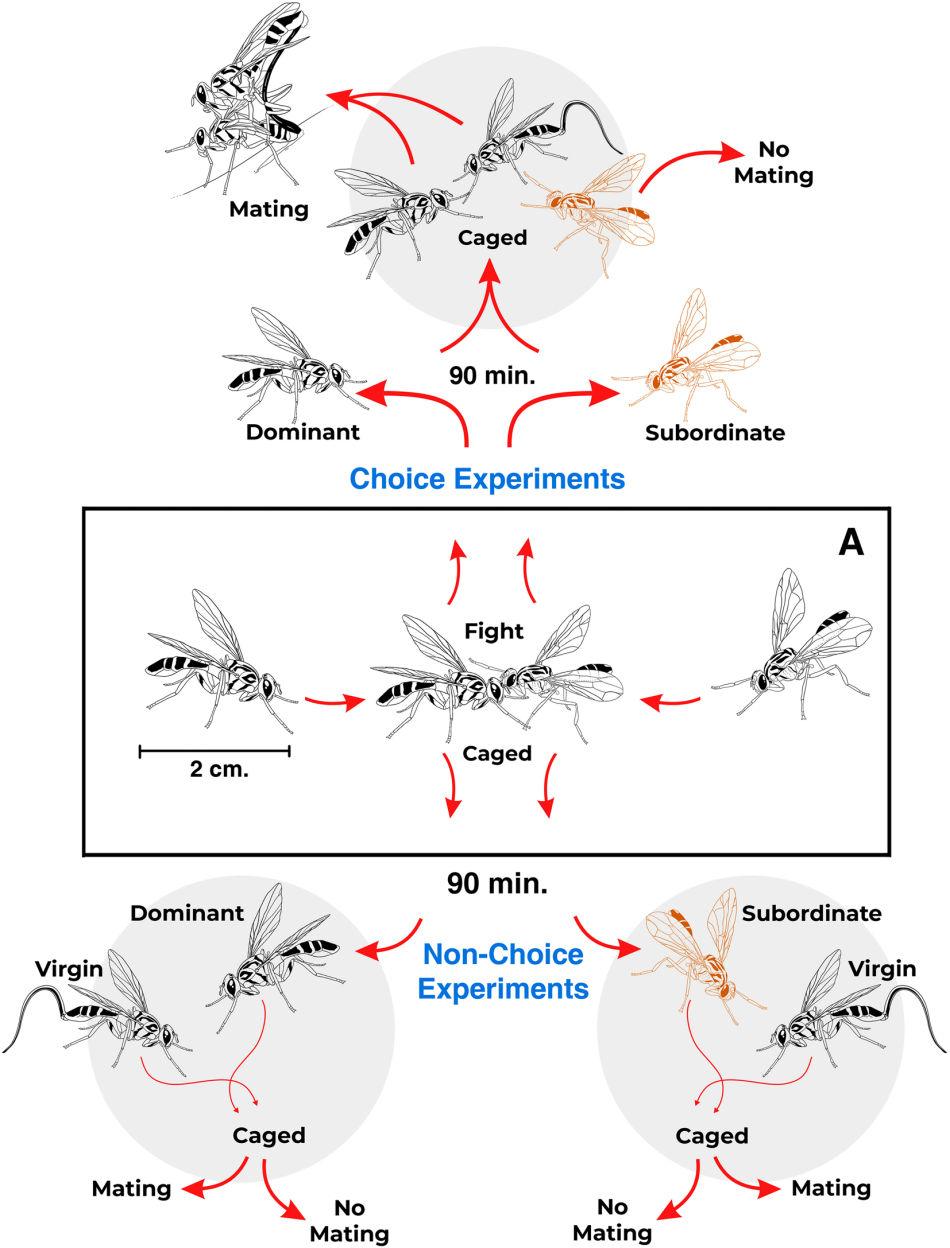
Experimental sequence for the establishment of dominance (hierarchy) among *Anastrepha curvicauda* males and subsequent mate choice and no-choice experiments. The experiment starts when two males of similar age and body weight were caged (A) and agonist behavior was quantified for 60 min. Ninety minutes after the hierarchy was established one male was classified as dominant and the other as subordinate (colored reddish, see text for details). The choice experiment (upwards in the sequence) involved three individuals: a dominant and a subordinate male (their hierarchy among them was established 90 min) and a virgin female caged for 60 min. The non-choice experiment involved 2 individuals: a male either dominant or subordinate and a virgin female caged for 60 min. In both experiments, the insects’ behavior was filmed and registered the male achieving the copula.

We tested the hypothesis that dominant male courting repertoire behavior and effort are affected by the presence or absence of a subordinate male. This was a two-stage experiment. In the first stage, we established whether a male was dominant or subordinate and, in the second stage we registered the sexual behavior of the dominant and subordinate male caged simultaneously or individually with a 5–7 days old virgin female and recorded which male was accepted by the female. See Fig. 7.

To establish the hierarchy among males, two males of 7–8 days old whose body weight was within ± 0.05 mg, were caged in a small plastic container (9 cm height × 3.5 cm diameter) for 60 min. Dominance was established according to the agonistic behaviors reported previously^47^ which may include threat (a male with the wings thigh to the body, raised abdomen and/or movement of the second pair of legs approaches the other male), attack (a male directly approaches the other male and touches him with the forelegs), fight (male responds the other male’s attack) and withdraw (a male goes away as a result of the agonistic behavior). Percentage of dominance was calculated as^48^:

$$ \% D = \left[ {n{1}/ \, \left( {n{1 } + n{2}} \right)} \right] \, * 100 $$

where: *%D* = percentage of dominance, *n*1 = No. of dominant contacts, *n*2 = no. of non-dominant contacts.

Dominant contacts were defined as any contact with any part of the body with the intention of harm to or displace the other male; this could be striking the head of the opponent with the forelegs, pushing with his body, etc. We classified males as dominant if %D > 70% or as subordinate if %D < 30%^48^. Only males over 70% and below 30% of dominance were used in the second stage of this experiment.

In preliminary experiments, we used different times (60, 90, and 120 min) to test the stability of the dominance-subordinate relationship among males. This time frame (60–120 min) was established according to the time that flies remain in the papaya groove and engaged in mating behavior. We found that once the hierarchy was established among males, this relationship did not change within 120 min. Ninety minutes after establishing the hierarchy of the males (dominant or subordinate) we performed two experiments: (1) a non-choice experiment where a 5–7 days old virgin female was caged with a dominant or a subordinate male, and (2) a choice experiment where two males (a dominant and a subordinate, who had previous contact with each other) were caged with a 5–7 days old virgin female (Fig. 7). Experiments were performed in the aforementioned cylindrical transparent acrylic tube. We tested 15 females for each type of experiment and recorded the male accepted by the female.

### Behavior description

#### Agonistic, sexual behavior, repertoire, and ethograms

To establish the agonistic behavior, focal and continuous 60 min observations of agonistic interactions^49^ between two males (n = 25) of similar age and weight were carried out in a transparent acrylic (20 cm diameter and 10.5 cm height) arena. Both males were released at the same time into the arena.

#### Ethograms and sexual behavior

The film of each experiment was reviewed to identify the different behaviors and behavioral elements displayed by the insects. With the frequencies of the observed behavioral elements, a first-order Markovian^50^ contingency table was constructed, and comprehensive ethograms were devised. This contingency table provided the frequency of transition from one behavioral element to all other elements. We used the behavioral elements reported previously^51,52^ for this fruit fly. To design the ethograms of each group of individuals, the median of each behavioral element was calculated. Only behavioral elements whose median was > 1 were included in the ethogram except on the choice experiments where the behavioral elements mounting, mating, and tandem were included because of their relevance to the male sexual behavior.

### Courtship variables

We registered: (1) Courtship Intensity Index—CII, the time the male invests courting the female. It was computed as CII = courtship duration/observation time; (2) Latency to courtship; defined as the time between the beginning of the observation session until the male heads for the female; (3) courtship length defined as the time (s) the male courts the female or remained with 10 cm of her without losing eye contact; (4) latency to mating defined as the time between the start of the experiment until male achieve mating and (5) percentage of mating define as the number of pairs achieving mating/total number of females tested.

### Statistical analysis

The percentage of patterns and transitions among courtship and the effect of hierarchy on mating behavior were analyzed with a Mann–Whitney test. A χ^2^ with Yates correction for continuity was used to analyze the percentage of mating. All analyses were carried out in SigmaPlot 12.5.
